# Mobile Health Apps to Facilitate Self-Care: A Qualitative Study of User Experiences

**DOI:** 10.1371/journal.pone.0156164

**Published:** 2016-05-23

**Authors:** Kevin Anderson, Oksana Burford, Lynne Emmerton

**Affiliations:** School of Pharmacy, Curtin University, Perth, Western Australia, Australia; University of Groningen, University Medical Center Groningen, NETHERLANDS

## Abstract

**Objective:**

Consumers are living longer, creating more pressure on the health system and increasing their requirement for self-care of chronic conditions. Despite rapidly-increasing numbers of mobile health applications (‘apps’) for consumers’ self-care, there is a paucity of research into consumer engagement with electronic self-monitoring. This paper presents a qualitative exploration of how health consumers use apps for health monitoring, their perceived benefits from use of health apps, and suggestions for improvement of health apps.

**Materials and Methods:**

‘Health app’ was defined as any commercially-available health or fitness app with capacity for self-monitoring. English-speaking consumers aged 18 years and older using any health app for self-monitoring were recruited for interview from the metropolitan area of Perth, Australia. The semi-structured interview guide comprised questions based on the Technology Acceptance Model, Health Information Technology Acceptance Model, and the Mobile Application Rating Scale, and is the only study to do so. These models also facilitated deductive thematic analysis of interview transcripts. Implicit and explicit responses not aligned to these models were analyzed inductively.

**Results:**

Twenty-two consumers (15 female, seven male) participated, 13 of whom were aged 26–35 years. Eighteen participants reported on apps used on iPhones. Apps were used to monitor diabetes, asthma, depression, celiac disease, blood pressure, chronic migraine, pain management, menstrual cycle irregularity, and fitness. Most were used approximately weekly for several minutes per session, and prior to meeting initial milestones, with significantly decreased usage thereafter. Deductive and inductive thematic analysis reduced the data to four dominant themes: engagement in use of the app; technical functionality of the app; ease of use and design features; and management of consumers’ data.

**Conclusions:**

The semi-structured interviews provided insight into usage, benefits and challenges of health monitoring using apps. Understanding the range of consumer experiences and expectations can inform design of health apps to encourage persistence in self-monitoring.

## Introduction

The increasing aging population will benefit from 21^st^ Century self-care techniques, easing burden on healthcare by enabling self-monitoring at home, office or other location.[1] In order for self-care of a chronic condition to be sustained, self-management techniques need to be integrated into one’s life.[2, 3] Due to differences between chronic conditions, there is no agreed definition of self-care.[4] One commonality is that self-care requires *self-monitoring* for a consumer to pursue daily decisions to maintain functionality.[4] Self-monitoring can be conducted by consumers on various levels; examples are self-awareness of symptoms (e.g. shortness of breath), manual blood pressure readings, and self-maintained electronic databases of blood glucose measurements in diabetes management. For consumers with reasonable health literacy, self-monitoring offers greater autonomy, aiming to reduce pressure on health resources.[5–8]

Despite being a relatively new phenomenon, self-monitoring has experienced notable developments in its practical immersion into one’s lifestyle. Health consumers are increasingly engaging with mobile health applications (‘apps’)[9] for self-monitoring. However, limited regulation in the technology marketplace enables insufficiently tested[10, 11] self-monitoring devices to be launched, with potential for health consumers to ill-advisedly change their self-care regimens. There are many instances of ‘buggy’ health apps.[10, 11] Indeed, a number of authors have called for guidelines around electronic self-monitoring to prevent errors or other incidents.[12, 13] In Australia, the introduction of the Health Market Validation Program[14] signifies the Victorian State Government’s and technology vendors’ commitment to remote/home monitoring; feasibility studies are required in other jurisdictions.

### Research incorporating Consumer Experience Metrics

One report, in which consumer experience with health apps was a key outcome, describes two Swiss university randomised pragmatic trials.[15] Both studies explored whether an app-based intervention was more effective than self-management of chronic pain without an app. The apps included modules for participants to write diary entries and complete questionnaires during the six-month intervention. Consumer experience was measured in terms of adaptability and pre-post sick leave,[15] with the Chronic Pain Acceptance Questionnaire[16] used to record sick leave taken by participants.

Consumer experience was also included in a four-week British weight management study involving seven females and six males.[17] A hybrid website and smartphone app were trialed. Semi-structured telephone interviews were used to assess the two platforms, with data analyzed via inductive thematic analysis.[17] Participants noted improvement in self-reported dietary and physical activity. No confounding factors relating to weight management were acknowledged. Key outcomes relating to goal engagement included motivation, self-efficacy, awareness, effort and achievement. The researchers encouraged critique of the app, whereby participants suggested use of barcode scanners and free-text entry boxes.

Since self-care transfers most of the responsibility to the consumer, the usability of technology for this purpose is imperative. Consequently, self-care technologies need to be adaptable to technological environments and user preferences.

A growing number of studies have explored the impact of technological interventions on consumers’ health outcomes. These interventions have included automated reminders (via text messaging)[18, 19] and internet-based information,[20] and have been assessed using self-report by participants,[21] with little, if any, external validation. Poor persistence with long-term self-monitoring is evident in chronic conditions such as asthma.[22] Gamification can be used to increase engagement through use of rewards for repeat logins within a period of time and achieved milestones.[23] With many usability features conceived to date, mobile health app design is constantly evolving;[24] many app development frameworks offer fast, scalable interfaces to deploy changes to user interfaces seamlessly.

An American health app study reported sociodemographic characteristics of app users, through a 36-item cross-sectional survey of 1604 English-speaking adults.[25] At least one health app had been downloaded by 934 of the participants. Data from open-ended questions, such as effectiveness of the app and reasons for halted use, were thematically analyzed by two researchers, and revealed Weight Loss, Calorie Tracking, Nutrition, and Physical Activity as their main themes. While facilitating statistical analysis, large-scale studies are compromised by their limited ability to probe participants for in-depth responses.

Studies into self-care using mobile apps have predominantly involved custom-designed apps. Examples are a pre-post intervention for asthma using the *Smart Phone Application*,[26] randomised-controlled trials for asthma using the *t+ Asthma* app[27] and another unnamed purpose-built app,[28] as well as a diabetes randomised-controlled trial using *Glucose Buddy*.[29] In these studies, self-efficacy was the only measurement of consumer experience, while participants’ engagement with the app was determined via self-report. Engagement does not necessarily mean long-term commitment to using the app; therefore, combining such data with usage statistics, such as login time and frequency and accessed features would add value to these studies. In contrast, mobile app-based obesity management in South Korea[30] applied the purpose-built *obesity-management app* constructed with ‘knowledge statements’ from an expert committee. Other custom-designed apps include an app for self-monitoring and guiding lifestyle management for breast cancer survivors[31] and PD Dr, a home-based monitoring assessment system for Parkinson’s disease.[32]

Notable deficiencies collectively demonstrated in these studies are their relatively short follow-up periods and lack of detailed consumer experience findings. Additionally, self-management programs have measured select outcomes, rather than a more holistic spectrum of outcomes relevant to conditions such as diabetes, osteoarthritis and hypertension.[4]

### Theoretical Frameworks

The Technology Acceptance Model (TAM), published in 1989, quantifies how consumers accept technology.[33] It is an extension of the Theory of Reasoned Action,[34] and is used to predict intended behaviour, adopting a technology-focussed paradigm in decision-making.[35] The TAM has been applied in qualitative[36] and quantitative[37] studies of health apps to determine the acceptance of mobile technology amongst physicians and medical students, respectively, and in health-related studies on topics such as adoption of health apps.[38]

The Health Information Technology Acceptance Model (HITAM) is an evolution of the third version of the TAM for the health technology field,[39] combining behavioural, personal, social and IT factors. This model also embraces the Health Belief Model[40] and has been used in asthma studies for investigation of medication compliance.[41, 42] However, no literature was found in which the HITAM informed research into the use of health apps.

The Mobile Application Rating Scale (MARS) is a validated and reliable scale[43] due to its internal consistency, inter-rater reliability and comprehensive extraction of 25 papers and resources in its formation. The MARS is an Australian development from 2015 to assess the quality of health apps, and is based on four quality scales: engagement, functionality, aesthetics and information quality. Research applying the MARS in studies involving health apps is emerging, with MARS noted in an Australian wellbeing evaluation protocol[44] and mentioned in an Irish mental health app feasibility study without being used in the study itself.[45]

Other theoretical models and frameworks have been applied in studies of self-care. The PRECEDE-PROCEED model has been applied in an asthma study in Taiwan[46] to measure factors such as asthma knowledge and self-efficacy; this model contains elements such as administrative and financial policies that may not be relevant to exploratory research.[47] Similarly, Orem’s Self-Care Model has been applied to asthma to investigate “self-care abilities, self-care practices, and health outcomes.”[48] However, this model and the PRECEDE-PROCEED model lack consideration of technological factors.

Review of the literature suggests the TAM, HITAM and MARS are the most relevant frameworks to qualitatively explore consumers’ experiences of mobile health apps. While no published research has applied a combination of these models, integration of the TAM, HITAM and MARS should improve cross-disciplinary relevance and robustness, and provide theoretical grounding for exploratory research into the consumer experience with health apps.

The objectives of this study were therefore to 1) qualitatively explore consumers’ experiences with mobile health apps and 2) their perceived benefits from use of health apps, and 3) formulate suggestions for improvement of health apps.

## Materials and Methods

This study explored consumers’ experiences with health apps through semi-structured interviews. The Human Research Ethics Committee of Curtin University approved this study (approval number RDHS-102-15). In accordance with this approval, participants provided signed informed consent for interview.

Inclusion criteria were minimum 18 years of age (no maximum), residence in the metropolitan area of the University (for convenience), conversational fluency in English, and recent (at least one month’s) experience with any health/fitness mobile app. Any duration of use of the app(s) was of value, because discontinued use and negative experiences complemented data from persistent users. It was intended to involve participants of a broad age range to combine experiences of the tech-savvy younger generation with the older generation.

Including fitness apps enabled participants with chronic conditions such as obesity, diabetes and high blood pressure to elaborate on their experiences without restricting them to disease-specific apps. Participants without a chronic condition were included to capture fitness app usage amongst chronic disease consumers and healthy counterparts.

Guiding the research was the post-positivism worldview where relationships can be reverse-engineered via tested approaches such as deductive analysis.[49] A reductionist philosophy was applied to deconstruct implicit and explicit responses into manageable variables. The qualitative paradigm was crucial to appreciate, observe and deduce consumers’ experiences. The use of individual interviews offered privacy, and enabled exploration of each user’s interaction with their identified health app(s). Semi-structured interviews provided participants freedom to elaborate on the interview guide (Table 1).

**Table 1:** Interview Guide.

Question	Elaboration Questions	Theory, study or construct
Which health app(s) have you used?	Do you still use that/those app(s)? (If multiple apps) Which of those apps are still on your device? Which of these do you still use? Which one(s) would you like to talk about today?	Experience
(If on present device) Please show me how you use your health app.	How did you set it up? What problems do you recall in setting it up? (Prompts: user interface, prompts, permissions, language used)	Technological literacy
For approximately how long have you used (did you use) this app?	How often do/did you use it? (If discontinued) Why did you stop using the app?	Experience
How did you ‘discover’ this app?	(Prompts: health prof recommendation, peer/family recommendation, self-search)	TAM—subjective norms[50]
On which platform do/did you use this app?	(Prompts: iPhone, iPad, Android phone, Android tablet)	Descriptors of use
What do/did you like about this app?	Does/did the app fulfil your needs? Why or why not? Do/did you enjoy sessions with your health app? How is/was working with your app satisfying? Is/was your health app worth recommending to others?	TAM—usefulness;[50] Mobile App Rating Scale[43]
How easy is/was using your app?	What makes/made the app information clear and understandable? How do/did you find the font size and representation? How do/did you add remarks to your readings?	TAM—ease of use;[50] Acceptance Factors of mobile apps[51]
Have you sometimes not known (did you sometimes not know) what to do next with your app?	Are/were there any parts of the app you don’t use, because they’re complicated? What app features do/did you find unintuitive? Do/did you sometimes wonder if you’re using the app the right way? Who do/would/did you turn to for help using the app (prompts: family, friends, or online forum)?	Technological literacy; Acceptance Factors of mobile apps[51]
Have you found any ‘bugs’ in your health app, or things it can’t do?	If the app crashes or freezes (crashed or froze), is/was it easy to restart? Have you ever given up due to technical glitches? Have you ever contacted the company about any technical glitches?	Limitations of the app; Acceptance Factors of mobile apps[51]
How much sight and sound stimulation do/did you get from your health app?	(Prompts: graphs, things that flash up, reminders about personal targets, warnings, sound effects/reminders)	Mobile App Rating Scale[43]
What customization features would you like to see in your health app?		Mobile App Rating Scale[43]
What is your view of information stored on the cloud?	Do you find it an invasion of privacy?	
Describe your Initial user profile setup	Was registration via social media e.g. Facebook, Google + an option?	
Is your health app affiliated with a government health organization?	(Researcher to check later if participant unsure)	Mobile App Rating Scale[43]
Does/did your doctor (or other main health care provider) know you have used this app?	(If yes) How would you describe his/her reaction? Are you encouraged by a health professional (pharmacist, general practitioner) to self-reflect on your chronic condition?	Doherty[52] Design and Evaluation Guidelines
What medical or technical jargon have you seen in your app which you don’t understand?		Doherty[52] Design and Evaluation Guidelines
Does your app use technology you are already familiar with?	Are the dialogue boxes and input fields similar to what you are used to?	Doherty[52] Design and Evaluation Guidelines
Do you feel you require a peripheral (plug-in or Bluetooth) device to operate your app more effectively?		Yin[53] Usability Risk Level Evaluation
Do you prefer tactile feedback (vibrations) over plain text feedback?	Have you noticed anything vibrate when you’ve done something wrong or you receive a warning?	Yin[53] Usability Risk Level Evaluation
What features of your app do you think conflict with each other?	(Prompt: inconsistent shortcuts)	Yin[53] Usability Risk Level Evaluation
Are you satisfied with the time taken to perform tasks on your app?	(Prompts: time to display graphs, time to synchronize information)	Yin[53] Usability Risk Level Evaluation
What age bracket are you?	18–25; 26–35; 36–45; 46–55; >55 years	
Your occupation?		
Your highest education?	Year 10 (junior high school); Year 12 (senior high school); TAFE (technical college); University	

Constructs of the TAM,[33] specifically, perceived ease of use and perceived usefulness, were included in the interview guide. The HITAM included constructs describing personal and social factors such as motivation, self-reflection, competition and recommendation. Additionally, features of the MARS, such as engagement and aesthetics, were included. Duplicated concepts between the three models were deleted. Questions were adapted to suit this study, with the interview guide comprising core questions and supplementary questions for clarification and elaboration. The supplementary questions reflected “acceptance factors of mobile apps,”[51] collectively capturing all perceivable aspects of consumers’ health app usage. Following independent review of the draft interview guide, the structure was revised to enable participants to reflect on more than one app during the interview.

Participants were a convenience sample of residents in the Perth metropolitan area, aged 18 years or older and conversationally fluent in English, who self-reported recent use of any commercially-available health/fitness app with capacity for self-monitoring and data input. Any duration of use was included, because discontinued use and negative experiences were considered to provide valuable insights into persistence, and complemented data from persistent users. No preferential sampling of participants with either negative or positive experiences was applied.

A multi-faceted recruitment strategy was applied. Participants were recruited via co-operation with health associations such as Celiac Australia and Diabetes Australia, through their social media accounts and eNewsletters over a period of four weeks. A local radio station popular with a mature demographic was also utilized in an attempt to recruit listeners with an interest in self-care. The first author’s affiliation with a technology start-up hub enabled a broadcast announcement to members to attract participants from different educational backgrounds who shared a common goal to innovate. A static text advertisement was posted on the University portal, in addition to posters with a Quick Response Code at shared university computer workspaces and the library.

Interviews were scheduled in quiet locations such as a public library or participant’s office. A single interviewer (author KA) conducted all interviews in June and July, 2015. The first several interviews were used to reflect on the question guide. Interviews were digitally recorded, supplemented with field notes and post-interview reflections. Data saturation was perceived through recurring explicit ideas[51] such as motivation, customisation, interconnectivity, data inaccuracy, convenience and competitiveness, and confirmed during analysis. Literature suggests 20–25 participants ranging generally provides adequate saturation of themes when using qualitative semi-structured interviews.[54–56] Advertising was halted after four weeks on-campus and seven weeks off-campus. Audio files were professionally transcribed by an accredited agency with privacy certification. Raw data files were imported to QSR NVivo 10 for open coding and analysis.

Braun and Clarke’s six-step thematic analysis approach was utilised to capture user experience themes,[57] addressing Objective 1. The deductive approach[58] was applied to continually reflect on, and refine, emerging themes. The deductive coding framework was synthesized using the TAM, HITAM and MARS. Step One involved data familiarization to verify accuracy of transcriptions. This required the researcher to become intimate with the transcripts by re-reading them and referring to field notes. As per Step Two from Braun and Clarke, co-authors confirmed selection of codes and themes, and amendments were made as necessary to reach consensus. Initial coding was performed in NVivo based on the deductive coding framework, with miscellaneous responses interpreted inductively into new codes as required. Two authors matched initial coding to ensure consistency and establish common ground to confirm definition of the full set of themes. Step Three involved clustering nodes to a common theme(s) based on coherent patterns. To ensure robustness, data extracts are quoted in the Results section to demonstrate legitimacy of the identified themes.[57] Step Four involved reduction of themes into most prevalent implicit and explicit ideas.[57] Redundant themes derived from the three published models were deleted. Step Five involved describing the parameters of, and naming, the themes, whilst Step Six involved reporting to convey the analysis made. Outcomes from Steps Four, Five and Six are reported in the Results.

Data are presented based on emergent themes from thematic analysis, exploring how health consumers use apps for health monitoring (addressing Objective 1). Perceived benefits from use of health apps (addressing Objective 2) and suggestions for improvement of health apps (addressing Objective 3) are presented descriptively.

## Results

### Description of Participants

The most common age bracket of participants was 26–35 years; one participant was over 50 years and another recently turned 18 years old; further participant demographics are provided in Table 2. Interviews were completed in 20 minutes on average, during which time, most participants answered all questions relevant to their experience.

**Table 2:** Participant Demographics.

Characteristic	Subcategory	Number
Gender	Female	15
	Male	7
Age (years)	18–25	4
	26–35	13
	36–45	2
	46–55	2
	>55	1
Total Participants	Met inclusion criteria	22
	Excluded	4
Interview Duration (minutes)	Mean interview time	20
	Shortest interview	15
	Longest interview	41
Smartphone Operating System	Android	7
	Apple	15
	Windows	0
	Symbian	0
	Linux	0
Main Language	English	17
	Other	5
Recruitment Source	Physical university posters and online staff/student portals	10
	National Asthma Council eNewsletter and social media (Facebook)	3
	Rare Diseases Australia eNewsletter	2
	Celiac Australia eNewsletter	2
	Diabetes Australia eNewsletter	1
	Perth start-up community (posters and daily notices blog)	4
	Pharmacy open 24/7	0
	Curtin University Radio	0
Occupation	Allied health	3 (podiatrist, psychologist and speech therapist)
	University student	5
	Other office-based workforce	9
	Retail	1
	Start-up innovator	4
Highest Education	High school	2
	University	18
	Other	2

Table 3 displays the types of apps reportedly used by the 22 participants, three of whom did not report any chronic condition. Nine apps were self-discovered, and two recommended by friends, four by a family member or partner, four by a healthcare professional and one by information from a health association or gym. The remaining two participants were influenced by multiple sources for different apps: self-discovery then a friend; and partner then a gym. All participants located their app using the respective app store on their smart device. For commercial reasons, the marketed names of the apps are not reported here. Persistence with each health app ranged from “a couple of weeks” for a diabetes app to “over two years” for a pain management app.

**Table 3:** Types of Health Apps used by Participants.

Type of App	Used by Android Participant Number	Used by iOS Participant Number	Number of Participants
Blood pressure monitoring app (1 type)		P6	1
Diabetes monitoring app (2 types)		P2, P17, P20	3
Migraine management app (2 types)		P5, P8	2
Menstrual cycle monitoring (4 types)	P1, P22	P6, P4	4
Anxiety management app (1 type)		P13	1
Calorie management and weight loss monitoring app (5 types)	P1	P2, P3, P16, P20	5
Celiac disease management app (1 type)	P11		1
Sleep monitoring app (4 types)	P14	P6, P13, P21,	4
Pain management app (2 types)		P8	1
Cycling app (2 types)		P12	1
Fitness App (22 types)	P8, P9, P11, P14, P18, P22	P2, P3, P7, P9, P10, P15, P16, P17, P19, P20, P21	17
Other (saliva analysis kit)		P16	1

The chronic conditions reported by participants included sleep disorders, chronic migraines, menstrual irregularities, chronic depression, arthritis and Behçet's disease. A number of participants reported more than one condition. Although the interviews focused on user experiences rather than their medical condition(s), participants were keen to share insights into their health as well as app usage.

One participant presented with the new Apple Watch^®^, seven participants presented with Android smartphones, and the remaining participants owned an iPhone 4, 5 or 6.

### User Experiences

Four emergent themes are described below, based on deductive analysis with reference to the TAM, HITAM and MARS. The themes were named Engagement, Functionality, Information Management, and Ease of Use. Each of the four themes aligned with constructs of one or more of the three published models.

### Engagement

Aligned with the MARS, the Engagement theme covers consumer interaction with their app, motivation to sustain usage, ability to self-reflect or write notes against readings, and social factors enabling competition with other users. Apps that can sustain positive behaviors and adapt to changes in consumer requirements were more likely to be used on a continual basis. This was particularly noted amongst users of pain, sleep and depression management apps. The following user of a blood pressure-monitoring app demonstrated persistence with his/her app:

*“I was diagnosed with high blood pressure* … *I've now been able to come off the medication*, *but I still monitor my blood pressure [documenting the readings into an app] just to make sure it's in a healthy range*.*”*

[P6]

Inability to engage with one’s health app can result in declined usage:

*“I do have some apps I don't use often*, *mainly because they've kind of bored me in a way*. *I'll just do an example*: *one fitness app shows you how to lose weight*, *but the way it's describing it*, *it's not what I'm after*. *It's one of those free apps I bought that—I thought [the fitness app] would be great*, *but when you actually use it*, *it's not the same*.*”*

[P2]

Most participants reduced or stopped using their app when they were familiar with how to self-manage and did not require constant interaction with their app. This finding was evident in users of strength training and fitness apps, whereby users who had reached their goal were not stimulated to achieve further, as well as the following user of a pain monitor:

*“I think the migraine one's probably outlived its usefulness for me*, *but the back pain one*, *I could still go back to that at any time*. *If I started to need to monitor my pain again in a systematic way*, *I'd still go back to it*. *But I haven't had back pain that's needed that*.*”*

[P8]

The same participant reported ‘outgrowing’ two pain-management apps:

*“So they've [migraine and pain tracking app apps have] sort of exceeded their usefulness now*, *but initially they were very helpful*. *Well*, *initially I was using them to track migraine symptoms and to track the effects of medication*. *But now I know what most of my triggers are*, *and I know what medication works*. *I guess for me to use it again*, *it would have to offer something different*. *So maybe alternative management strategies to what I'm already doing*.*”*

[P8]

Convenience was found to be the main factor why participants engage with health apps, as exemplified by a participant who used a smartwatch app for weight management:

*“I really want to have a more active lifestyle* … *Being able to just look at [the smartwatch] on the fly and going*, *‘Right*, *if it just means that I have to go move that little bit more*, *or I have to exercise that little bit more’*, *I will do it*, *because you have a real-time gauge of how well you've done for the day*. *So that gets me going because the perceived barrier of just getting the thing done is a lot lower*.*”*

[P7]

With one exception [P8], all reported increased engagement when describing the social or competitive angle of their app by all participants. This phenomenon was noted for fitness trackers over other health monitors. Examples were:

*“Yes*, *thankfully [I am socially competitive]*. *Bragging rights*, *unfortunately*, *count*.*”*

[P9]

*“Whenever we do a weekend challenge*, *you always have a look at what the other person's doing and [their] competitive side*. *I just want to beat the other people I see on there*, *so [using the app] is quite a good motivator*.*”*

[P10]

Having to purchase an app was expected to increase users’ engagement and persistence with the app. Two cases relating to this concept were of note. Participant 5 had experienced migraines for over 20 years, and used a migraine app for one year after recommendation from her neurologist who suggested using a migraine diary. Participant 16 was an app developer who used an app for weight management. Neither expressed concern paying for health apps:

*“Usually apps are not expensive*, *they're usually under $5*. *So if you found something really good*, *you would definitely pay*.*”*

[P5]

*“I'm prepared to pay for applications*. *As well as being in the software industry*, *I understand that it's people's livelihoods are attached to this*. *I use some free applications*, *but I often will pay for the upgraded or the purchased option*.*”*

[P16]

### Functionality

Aligned with the MARS and HITAM, Functionality encompasses guidance provided by the app developers, aesthetics, annoying features, layout, navigation and tactile feedback. While Functionality and Engagement are subjective concepts, Functionality relates to more tangible physical features. One consumer found color-coding in outputs a useful function:

*“These pink patches are REM [rapid eye movement] sleep*. *The green*, *which is the light sleep*, *is basically stage two sleep*. *The blue is your stage three*, *four sleep*.*”*

[P13]

Use of tactile, visual and sound feedback was divided amongst participants, based on consumer preference and task performed:

*“Yeah [I haven't disabled the auditory alerts]*. *My running app will ping every so often* … *saying a friend has completed a run*, *or it's time for me to do a run*, *or something along those lines*. *[My app with a wristband device] sends me a little alert if I'm close to my goals*, *if I've got 2*,*000 more steps to go*. *[The auditory alert] doesn't really bother me*. *I just tune out*.*”*

[P9]

*“I would find that annoying*, *yeah [push notification suggesting exercise]*. *I'd turn it off*.*”*

[P10]

App functionality is dependent on the environment in which it is used. For example, a participant using a cycling app did not use any tactile or sound feedback:

*“I usually keep it [the smartphone with the cycling app] on my bike while I'm riding*, *so I can see the speed*, *and the time*, *and distance and things*. *I don't think I use any sound or anything like that*.*”*

[P12]

Reminders to upgrade app versions for greater functionality were deemed annoying:

*“With [the weight management app]*, *they always ask you to upgrade to Pro*, *so you get more advice and stuff*, *but that's really annoying*.*”*

[P1]

When asked about peripheral devices to synchronize with a diabetes app, a participant responded:

“*That would be very helpful*, *yes*.*”*

[P2]

Despite well-received navigation and layout features, the physical requirements for apps to measure sleep duration and quality were inconvenient:

*“You have to put it [the phone] under your sheet*, *on the mattress*, *or under your pillow*, *and I think I just always had that consciousness that my phone was there and I had to remember to turn [the app] on before I went to sleep and turn it off again when I woke up*, *and it just wasn't really contributing to good sleep hygiene*.*”*

[P6]

Some participants indicated inclination towards customizing app features to suit their requirements:

*“I would love* … *to be able to record reps*, *and sets*, *and weights and things like that [if their running app were more customisable]*.*”*

[P3]

### Information Management

Information Management is aligned with the HITAM, and describes reliability, privacy to third parties, data security at rest and in transit, and quality and quantity of data. Without acceptable information management processes, health apps would lack the ability to compute readings, analyse data accurately, reject false or faulty entries and securely manage data. Data security appeared highly valued by participants, but was generally dependent on the type of data. For example, self-documenting height and weight did not raise any concern, although concerns were raised around access to those data by health insurers. One participant [P8], who used a sleep management app, expressed some concerns about potential access to stored data. Another [P13] had created a separate account for services used to preserve privacy. A user of a menstrual cycle tracker [P4] was not comfortable with the prospect of her data accessed by third parties, while another was less concerned:

*“I don't think about [data security]*, *to be honest*. *This is going to sound terrible—maybe I'm just really naive* … *I don't know*, *it doesn't really concern me*. *Probably*, *it should*.*”*

[P22]

Similarly, the following participant did not have any significant concerns about data security relating to a fitness tracking app:

*“Not in this instance [no concerns about cloud storage]*. *I think there's been a lot of hoo-ha about it*. *And this is a company; I've been with Apple for a long time*. *They've done a good job making consumers feel that their data is safe* … *for Apple*, *because the data is just used for the benefit of the consumer*. *It's not otherwise; I have no qualms about it*.*”*

[P7]

Counterbalanced against privacy issues, there was some gravitation towards apps interconnected with consumers’ healthcare services, as one participant with chronic migraine explained:

*“I think it's not so much the app*, *but it's where the app can go*… *If it's just an app in isolation*, *it doesn't have as much power [compared to] if it's something that you can feed into information that you need somewhere else*.*”*

[P5]

Apps interconnected with each other were also of interest:

*“Yes*, *that would work*. *I don't mind that*. *[if her diabetes app was connected to an insurance provider]*.*”*

[P17]

The ability to send blood pressure readings to general practitioners for analysis was highly valued by a participant:

*"What I really liked about the blood pressure app is that it's really easy for me to export my results and email them* … *and I've actually done that before for the doctor*. *He said*, *‘How are you tracking with your blood pressure*?*’ I've just been over [to the clinic]*. *While I'm sitting there and the doctor's in clinic*, *[I] just email him a PDF straight away of my results*. *He's able to save that on his computer*, *so it's quite handy for him too*.*”*

[P6]

Glitches in accuracy were reported in some apps:

*“I was actually overseas back in April*, *and for some reason*, *[the wearable device] keeps on syncing back to the time when I was Turkey* … *which is a bit inaccurate*.*”*

[P2]

Additionally, the ubiquitous nature of self-care apps captured all forms of movement, at times leading to glitches:

*“I went go-karting a while ago and [the app] thought I did like a hundred flights of stairs and thousands and thousands of steps in the hour I was driving around* … *I know it's never going to be exact*, *but if it's within a few hundred steps*, *then that's fine*.*”*

[P10]

The following consumer was familiar with environments instigating inaccurate heart rate readings, and was able to rectify the issue:

*“Sometimes [the heart rate app] gives numbers that are definitely not right*, *and then I'm like*, *"Okay*, *the lighting was too low" and discard that*. *I've noticed when* … *you're really cold*, *or if the florescent lighting is coming on a funny angle that [the phone's camera] will sometimes not register that there's too little lighting*, *or that the situation isn't going to give a good [heart rate] reading*. *So I tend to do it [measure heart rate using the app] twice rather than once*.*”*

[P13]

Some participants were particularly keen on statistics, and utilized their data in a more sophisticated way than others who merely glanced at their graphs and charts:

*“I think I'm the sort of person that I like to see the data around whatever problem I've got*, *just to help me understand it and monitor it*. *So I'm always really interested in seeing the statistic*.*”*

[P6]

*“For me*, *the major interest was the ability to export my data and consume it*, *and interpret it*, *and analyse it in a set of third-party tools*… *“I use some of our heavier statistical analysis tools from work against the number of times I go running and get some insight there*.*”*

[P16]

The same participant [P16] particularly valued using existing phone hardware to measure heart rate and blood pressure:

*“So this technology is a really interesting use of the phone*. *Obviously*, *the camera flash*, *and the camera*, *and the light weren't intended for that use [heart rate*, *blood pressure using the smartphone’s flash and camera]*. *I quite like that an entrepreneur somewhere has seen that these pieces of technology can be used to create something different* … *I would be interested more in things like blood pressure and even* … *blood glucose levels*, *and some of the measurements which I suspect are probably useful for people with diabetes and what have you*.*”*

[P16]

### Ease of Use

Ease of Use is aligned with the TAM, and includes concepts such as automation, convenience, fun and health literacy suitable to cater a range of consumers. Recurring patterns among the 22 participants included the desire to use the app, particularly until consumers had reached their self-management goal. Various app features were appreciated by consumers, for example:

*“The audio cues [telling me my duration and distance on my running app* … *I really like them*.*”*

[P3]

Automation of in-app functions reduces time to perform tasks and was appreciated by all participants:

*“I use* … *[certain health apps] because they're connected to wearables*, *so I don't have to do a lot of the data collection*. *It does it automatically for me and then feeds me back the information*.*”*

[P9]

Convenience was appreciated by one user in self-managing diabetes:

*“This one's quite good because it helps you out with planning*. *It also has information on how you can upload what your blood sugar levels are like*. *And it even shows you meal-time goals*, *it shows you how much juice you can have*, *how many starchy foods*, *how much protein*, *but even suggests what you should have every day*, *which is helpful*, *and it shows you what you can do*, *because you may think*, *‘Oh*, *well*, *eight pieces of vegetables is a lot*,*’ but when you look into it*, *it's not that much*, *really*.*”*

[P2]

#### Perceived Benefits

The analysis in this section represents the benefits of using a health app to facilitate self-care, analysed inductively from coded interview data.

Perceived benefits from usage of health apps included greater self-awareness of one’s condition, easier integration of self-management in daily life, ability to send data to allied health professionals without repeated visits, the ability to view historical data without visiting a doctor, social motivation to improve fitness, and desire to customise app features to suit individual needs. Participants also expressed greater control of their condition, in this case, menstrual problems:

*“I decided just to search and find out whether there was an appropriate app just to make life a little bit easier*… *my specialist had told me to keep track of any symptoms and the length of my cycle*, *so I just found [the menstrual cycle tracker] myself online*, *and found that to be an easy tool to use*.*”*

[P6]

#### Suggestions for Improvement

Suggestions for improvement were identified deductively from responses, and were not constructs of the TAM, HITAM or MARS. Suggestions included:

*“[The diabetes app] could remind you when to do your blood sugar*, *say*, *before your meal or two hours after your meal*… *would be very helpful*, *because that's another thing I get amnesia on*, *is forgetting about [when to take blood sugar readings]*. *A beep would help me*, *but being at work*… *I'll turn my phone on silent—a vibration would be helpful just to remind you*.*”*

[P2]

*“Maybe if I could leave the features I don't use [in this menstrual cycle tracker] behind*, *since I'm not trying to get pregnant*, *so just get rid of these fertile days*.*”*

[P4]

*“[Receiving benefits for sharing migraine management tips with other app users] would be amazing* … *if I could have just pressed a button and sent it in [my migraine action plan from the app to the doctor’s email]*, *that would have been ideal*.*”*

[P5]

The aforementioned limitation about fitness-tracking apps not recognizing certain activities was also mentioned by another participant, who suggested:

*“I guess being able to track different styles of exercise*, *so not just running and cardio-based activities*, *but if it could somehow track better movement with the bodyweight exercises or high-intensity exercises*, *which aren't as cardio-based*.*”*

[P3]

Furthermore, the same participant [P3] gravitated towards more interconnectivity of raw data from Medicare and data from multiple apps aggregated in one graph. Suggestions for improvement included appropriate use of gamification techniques throughout the app.

## Discussion

### Principal Findings

Data from this limited sample of health app users suggest self-management by health consumers with chronic conditions can be enhanced via use of mobile applications. This is the first-known research to combine these models, benefits of which include chronic condition-specific dimensions such as targeting health and information technology literacy, as well as functionality, engagement and information management. Additionally, more depth identifying usability issues when exploring consumer interaction with self-management goals via health apps was encountered when combining these three models. While the TAM and HITAM were not developed specifically for mobile apps, combining it with the MARS enabled a targeted, mobile health app focus and backing from more established technology acceptance constructs. Combining the TAM and HITAM with the more-recently-published MARS also provides an updated framework to assess health app usability. As confirmed by one study, health behavior is too complex and multi-faceted for one model to cover comprehensively,[39] which is why relevant constructs from TAM, HITAM and MARS were combined.

Similar qualitative studies include user perception of an oral health app.[59] However, user responses in that study were gathered via an online survey with no follow-up questions. Another health app study measured spirometry readings from adolescents with asthma and had no qualitative component.[60] This is the first study to explore self-care consumer experiences with health apps amongst adults. Our study covers a broader range of health apps, and more depth in exploring consumers’ experiences.

Randomised-controlled trials have reported clinical impact of health apps on outcomes such as self-efficacy, but have not focused on consumer interaction and engagement. No controlled trials have been published exploring consumer engagement with health apps. Adopting a qualitative approach has enabled insight into consumers’ experiences with health apps across a range of health conditions and with sufficient depth to understand motivators, desired features and issues relating to persistence.

The MARS was designed to provide quality star ratings for health apps.[43] This research has aligned the ‘Engagement’ theme from the MARS in the context of health apps. ‘Functionality’, concerning the operability of apps, is aligned with the MARS and HITAM, the HITAM introducing concepts such as health beliefs. ‘Information Management’ was aligned from the HITAM, while ‘Ease of Use’ was aligned from the TAM and relates to personalization of the user experience. This research provides novel insight from combined models to describe the experiences of users of health apps. User experience design considers user experience, including usability and perceived enjoyment of the product.[33, 61]

This study has established that self-management of a chronic condition using an app requires constant stimulation to accommodate changing user requirements and changes in wellbeing. Additionally, this study determined consumers with chronic conditions such as diabetes, depression, weight and sleep management issues are often recommended fitness apps. There were participants with a chronic condition who only used a fitness app and no disease-specific app. During the interview, no participant expressed confusion using an app to the point additional training or information such as on YouTube^®^ was required.

The benefits of gamification in health apps have been reported,[23, 62] but there has been only one chronic disease clinical trial using gamification, amongst minors.[60] Gamification in a health context does not necessarily ‘trivialize’ health management.[23] However, gamification involving inter-personal competition would be most suited to fitness trackers, whilst gamification for health apps would be most suited to intra-personal competition. Some apps were identified by participants as incorporating elements of gamification,[62] and provided those participants dynamic opportunities to engage with their health apps, such as receiving badges, passing levels and animated learning.[23]

Some health apps are designed for novelty or entertainment purposes, such as those providing blood pressure readings via touching the screen;[63] the accuracy of such outputs for medical monitoring is questionable. All participants presented apps designed for the intended medical or health purpose. Research suggests a paucity of evidence-based apps.[43] Restricting our inclusion criteria to evidence-based apps would have been inefficient, since research in this area is regularly updated.

Apps used via smartwatches and mobile telephones should offer more convenience than those requiring a tablet device or personal computer. This was confirmed by the participant who presented with an Apple Watch^®^ for convenient use of the health app of choice. As consumer uptake of smartwatches and other smart wearables increases, unique functionality with apps will emerge.

The present data cannot conclusively support the correlation between “willingness to pay” and “user experience”, although the correlation has been reported elsewhere in a study of mobile apps.[61]

Actual health benefits from engagement with self-monitoring can only be determined through clinical trial of an app. Nevertheless, *perceived* benefits from self-documentation can improve a consumer’s engagement with a health app, and provided the measurements are valid and reliable, this practice would presumably improve self-management. Positive oral hygiene self-management have been reported as a result of engagement with a health app.[16]

Some participants indicated their health professionals (dietician, psychologist or general practitioner) are already receiving consumers’ self-reported data electronically. How health professionals use these data requires further investigation, specifically, whether they cross-check consumer-reported clinical readings with their own, or consider trends in consumer-reported data.

Participants tended to reduce usage of their app when they reached their goals and no new self-management techniques were offered. For app engagement to be sustained after reaching a goal or for usage to become habitual, regular intervals of engagement are recommended. Rewards for chronic conditions involve intra-personal competition and involve different metrics to fitness apps employing more persistent and active inter-personal competition. Fitness apps can be used by consumers with chronic conditions such as diabetes and depression as part of a self-management program. At present, there is no research exemplifying long-term impact of reward-based engagement for mobile health apps.

Health monitoring devices are steadily increasing in market availability, with biometric-based innovations reducing the need for manual data input by consumers and providing more advanced, ubiquitous features.[25] Partnerships between health researchers and start-up communities, known for their agile coding methods, could help develop health apps conformant with the themes identified in this research: Engagement, Functionality, Information Management and Ease of Use.

### Strengths and Limitations

As explained previously, strengths of this study include combining the TAM, HITAM and MARS in a single study, which has not been attempted before, providing greater breadth in the deductive analytical framework than with the use of a single model. Additionally, using the post-positivism paradigm supports the concept of ever-changing consumer user requirements by viewing “knowledge as conjectural.”[64]

Limitations in this study include not referring participants to suitable apps based on their insight, and not scheduling a follow-up interview to gauge a change in their user experience. As such, these data represent a point-in-time measurement, and longitudinal research would better gauge individuals’ changes in self-monitoring patterns. This study was limited to a predominantly tertiary-educated Australian perspective; apps marketed internationally may incorporate different user experience metrics. This study did not quantify participants’ experiences, which would be of greater use and relevance when a single app is studied. It is unknown whether male and female users of health apps differ in their usage and expectations of these apps. The present sample comprised mostly female participants, possibly due to the recruitment methods.

This study is unable to correlate user experiences with credibility of health app. It may be possible for users to report positive experiences with an app that lacks an evidence base; conversely, an evidence-based app might be poorly designed, with low levels of engagement or usability. There are minimum design guidelines for the Apple App Store^®^[65] and similar guidelines for Google’s Play Store^®^,[66] although these were not assessed in our study.

Our research has revealed a range of apps used by consumers with a particular health condition, and use of multiple health apps. It would not be feasible to focus the study on one app; this would also limit the generalizability of the findings.

This study deliberately included a broad range of users of a variety of health apps, and it is not feasible to draw correlations or associations between groups of participants. Because some consumers used more than one app to manage their condition, any attempt to document the outcomes from use of a particular app could be confounded, and would rely on self-report. Evaluation of the clinical contribution of apps to health care requires careful experimental design and control of environmental influences on self-management of the medical condition of interest.

Participants discussed the app with which they are most familiar (most engaged), as this would highlight any frustrations they had encountered with programming bugs and limitations. However, participants were welcome to discuss other health/fitness apps with which they had experience. In the interests of keeping participants engaged in the interview, and ensuring currency and validity of the data, it was not considered worthwhile for participants to discuss all health/fitness apps they recalled using.

### Further Research

Future research may focus on users of apps for a particular health condition (e.g. asthma), with longitudinal monitoring of their engagement with a selected app(s) and changes in user experiences. Usage of apps incorporating gamification is an area requiring supplementary research, to enable researchers to gauge whether artificial intelligence has been designed in an intuitive and compatible way with regard to consumers’ health objectives.

The concept of competitive wellbeing also warrants consideration, with social Application Programming Interfaces linking health data to social media and other services to increase motivation and competitive spirit, and to assist users to achieve health goals.[67] Chronic conditions require persistent self-management and longitudinal monitoring, and health apps should deliver a customized solution for the user’s condition.[68] Moreover, sustained use of apps can be optimised by further insights into how consumers use apps.[69]

## Conclusion

This study explored consumers’ interactions with health apps through semi-structured interviews, uncovering a wide variety of users with a degree of commonality in their user experiences. User experiences have been described via four themes: Engagement, Functionality, Information Management and Ease of Use. These themes describe concepts such as motivation, customization, interconnectivity, data inaccuracy, convenience and competitiveness, and suggest how health apps can benefit by ‘growing’ with the user and adapting to changing operating environments.

## References

[1] Wiederhold BK, Riva G, Graffigna G. Ensuring the best care for our increasing aging population: health engagement and positive technology can help patients achieve a more active role in future healthcare. Cyberpsychol Behav Soc Netw. 2013;16(6):411–12. 10.1089/cyber.2013.1520 23751102

[2] Lorig KR, Sobel DS, Stewart AL, Brown BW Jr, Bandura A, Ritter P, et al Evidence suggesting that a chronic disease self-management program can improve health status while reducing hospitalization: a randomized trial. Med Care. 1999;37(1):5–14. 10413387 10.1097/00005650-199901000-00003

[3] Holman H, Lorig K. Patient self-management: a key to effectiveness and efficiency in care of chronic disease. Public Health Rep. 2004;119(3):239 15158102 10.1016/j.phr.2004.04.002 PMC1497631

[4] Chodosh J, Morton SC, Mojica W, Maglione M, Suttorp MJ, Hilton L, et al Meta-analysis: chronic disease self-management programs for older adults. Ann Intern Med. 2005;143(6):427–38. 16172441 10.7326/0003-4819-143-6-200509200-00007

[5] Williams MV, Baker DW, Honig EG, Lee TM, Nowlan A. Inadequate literacy is a barrier to asthma knowledge and self-care. Chest. 1998;114(4):1008–15. . 9792569 10.1378/chest.114.4.1008

[6] Baker DW, Gazmararian JA, Williams MV, Scott T, Parker RM, Green D, et al Functional health literacy and the risk of hospital admission among Medicare managed care enrollees. Am J Public Health. 2002;92(8):1278–83. . 12144984 10.2105/ajph.92.8.1278 PMC1447230

[7] Baker DW, Parker RM, Williams MV, Clark WS. Health literacy and the risk of hospital admission. J Gen Intern Med. 1998;13(12):791–8. . 9844076 10.1046/j.1525-1497.1998.00242.x PMC1497036

[8] Williams MV, Parker RM, Baker DW, Parikh NS, Pitkin K, Coates WC, et al Inadequate functional health literacy among patients at two public hospitals. JAMA. 1995;274(21):1677–82. . 7474271

[9] Gill PS, Kamath A, Gill TS. Distraction: an assessment of smartphone usage in health care work settings. Risk Manag Healthc Policy. 2012;5(1):105–14. 22969308 10.2147/RMHP.S34813 PMC3437811

[10] Dumas RA. Health App completely buggy? [Internet]. c2014. Available: https://discussions.apple.com/thread/6680914. Accessed 2 November 2015.

[11] Thomas O. Apple's health app is an embarrassment [Internet]. c2014. Available: http://readwrite.com/2014/10/02/apple-health-app. Accessed 2 November 2015.

[12] Finkelstein EA, Chay J, Bajpai S. The economic burden of self-reported and undiagnosed cardiovascular diseases and diabetes on Indonesian households. PLoS ONE. 2014;9(6):e99572 10.1371/journal.pone.0099572 24915510 PMC4051736

[13] Miller KM, Beck RW, Bergenstal RM, Goland RS, Haller MJ, McGill JB, et al Evidence of a strong association between frequency of self-monitoring of blood glucose and hemoglobin A1c levels in T1D exchange clinic registry participants. Diabetes Care. 2013;36(7):2009–14. 10.2337/dc12-1770 . 23378621 PMC3687326

[14] Host T, Person C, Lewis P. Health Market Validation Program (Health MVP) call for proposal application form for SMEs. [Internet]. c2014. Available: http://www.business.vic.gov.au/grants-and-assistance/programs/health-market-validation-program. Accessed 20 February 2015.

[15] Blödt S, Pach D, Roll S, Witt CM. Effectiveness of app-based relaxation for patients with chronic low back pain (RelaxBack) and chronic neck pain (RelaxNeck): study protocol for two randomized pragmatic trials. Trials. 2014;15(1):490–99. 10.1186/1745-6215-15-490 25511185 PMC4301893

[16] Nilges P, Köster B, Schmidt CO. Pain acceptance—concept and validation of a German version of the chronic pain acceptance questionnaire. Schmerz. 2007;21(1):57–8. 17111168 10.1007/s00482-006-0508-1

[17] Morrison LG, Hargood C, Lin SX, Dennison L, Joseph J, Hughes S, et al Understanding usage of a hybrid website and smartphone app for weight management: a mixed-methods study. J Med Internet Res. 2014;16(10):e201 10.2196/jmir.3579 PMC4259922. 25355131 PMC4259922

[18] Cooper S, Foster K, Naughton F, Leonardi-Bee J, Sutton S, Ussher M, et al Pilot study to evaluate a tailored text message intervention for pregnant smokers (MiQuit): study protocol for a randomised controlled trial. Trials. 2015;16(1):s13063-014-0546-4. 10.1186/s13063-014-0546-4 ; PubMed Central PMCID: PMCPmc4318454. 25622639 PMC4318454

[19] Haug S, Castro RP, Filler A, Kowatsch T, Fleisch E, Schaub MP. Efficacy of an Internet and SMS-based integrated smoking cessation and alcohol intervention for smoking cessation in young people: study protocol of a two-arm cluster randomised controlled trial. BMC Public Health. 2014;14(1):1140–48. 10.1186/1471-2458-14-1140 ; PubMed Central PMCID: PMCPmc4228117. 25369857 PMC4228117

[20] Proudfoot J, Clarke J, Birch M, Whitton AE, Parker G, Manicavasagar V, et al Impact of a mobile phone and web program on symptom and functional outcomes for people with mild-to-moderate depression, anxiety and stress: a randomised controlled trial. BMC Psychiatry. 2013;13(1):312–24. 10.1186/1471-244X-13-312 24237617 PMC4225666

[21] Eyles H, McLean R, Neal B, Doughty R, Jiang Y, Mhurchu C. Using mobile technology to support lower-salt food choices for people with cardiovascular disease: protocol for the SaltSwitch randomized controlled trial. BMC Public Health. 2014;14(1):950–8. 10.1186/1471-2458-14-950 . 25217039 PMC4180840

[22] Hasford J, Uricher J, Tauscher M, Bramlage P, Virchow JC. Persistence with asthma treatment is low in Germany especially for controller medication—a population based study of 483051 patients. Allergy. 2010;65(3):347–54. 19712117 10.1111/j.1398-9995.2009.02161.x

[23] Zichermann G. Gamification by design: implementing game mechanics in web and mobile apps. Cunningham C, editor. Sebastopol: O'Reilly Media; 2011.

[24] Pandey A, Hasan S, Dubey D, Sarangi S. Smartphone apps as a source of cancer information: changing trends in health information-seeking behavior. J Cancer Educ. 2013;28(1):138–42. 10.1007/s13187-012-0446-9 23275239

[25] Krebs P D D. Health app use among US mobile phone owners: a national survey. JMIR mHealth uHealth. 2015;3(4):e101 10.2196/mhealth.4924 26537656 PMC4704953

[26] Licskai CJ, Sands T, Ferrone M. Development and pilot testing of a mobile health solution for asthma self-management: Asthma action plan smartphone application pilot study. Can Respir J. 2013;20(4):301–6. 23936890 10.1155/2013/906710 PMC3956342

[27] Ryan D, Price D, Musgrave SD, Malhotra S, Lee AJ, Ayansina D, et al Clinical and cost effectiveness of mobile phone supported self monitoring of asthma: multicentre randomised controlled trial. BMJ. 2012;296(6614):e1756 10.1136/bmj.e1756 PMC3311462 22446569

[28] Liu WT, Huang CD, Wang CH, Lee KY, Lin SM, Kuo HP. A mobile telephone-based interactive self-care system improves asthma control. Eur Respir J. 2011;37(2):310–7. 10.1183/09031936.00000810 . 20562122

[29] Kirwan M, Vandelanotte C, Fenning A, Duncan M. Diabetes self-management smartphone application for adults with type 1 diabetes: randomized controlled trial. J Med Internet Res. 2013;15(11):e235 10.2196/jmir.2588 . 24225149 PMC3841374

[30] Jeon E, Park H. Development of a smartphone application for clinical-guideline-based obesity management. Healthc Inform Res. 2015;21(1):10–20. 10.4258/hir.2015.21.1.10 25705553 PMC4330194

[31] McCarroll ML, Armbruster S, Pohle-Krauza RJ, Lyzen AM, Min S, Nash DW, et al Feasibility of a lifestyle intervention for overweight/obese endometrial and breast cancer survivors using an interactive mobile application. Gynecol Oncol. 2015;137(3):508–15. 10.1016/j.ygyno.2014.12.025 WOS:000355779000025. 25681782

[32] Pan D, Dhall R, Lieberman A, Petitti DB. A mobile cloud-based parkinson’s disease assessment system for home-based monitoring. JMIR mHealth uHealth. 2015;3(1). 10.2196/mhealth.3956 PMC4392174 25830687

[33] Davis FD. Perceived usefulness, perceived ease of use, and user acceptance of information technology. MIS Quart. 1989;13(3):319–40.

[34] Madden TJ, Ellen PS, Ajzen I. A comparison of the theory of planned behavior and the theory of reasoned action. Pers Soc Psychol B. 1992;18(1):3–9. 10.1177/0146167292181001 WOS:A1992HC29000001.

[35] Fishbein M, Ajzen I, Albarracin D, Hornik RC. Prediction and change of health behavior: Applying the reasoned action approach. Mahwah: Lawrence Erlbaum Associates; 2007.

[36] Yarbrough AK, Smith TB. Technology acceptance among physicians: a new take on TAM. Med Care Res Rev. 2007;64(6):650–52. 17717378 10.1177/1077558707305942

[37] Briz-Ponce L, García-Peñalvo F. An empirical assessment of a technology acceptance model for apps in medical education. J Med Syst. 2015;39(11):1–5. 10.1007/s10916-015-0352-x 26411928

[38] Cho J, Quinlan MM, Park D, Noh GY. Determinants of adoption of smartphone health apps among college students. Am J Health Behav. 2014;38(6):860–70. Epub 2014/09/11. 10.5993/ajhb.38.6.8 . 25207512

[39] Kim J, Park H-A. Development of a health information technology acceptance model using consumers' health behavior intention. J Med Internet Res. 2012;14(5):e133 10.2196/jmir.2143 23026508 PMC3510715

[40] Janz N, Becker M. The health belief model: a decade later. Health Educ Behav. 1984;11(1):1–47. 10.1177/109019818401100101 6392204

[41] Becker MH, Radius SM, Rosenstock IM, Drachman RH, Schuberth KC, Teets KC. Compliance with a medical regimen for asthma: a test of the health belief model. Public Health Rep. 1978;93(3):268–77. 652949 PMC1431906

[42] Cormier DJ, Ferreira DC, Vise KM, Cahalin LP. A pilot study of childhood health behaviour and asthma using the health belief model. J Cardiopulm Rehabil. 2006;26(4):250–74. 10.1097/00008483-200607000-00015

[43] Stoyanov SR, Hides L, Kavanagh DJ, Zelenko O, Tjondronegoro D, Mani M. Mobile app rating scale: a new tool for assessing the quality of health mobile apps. JMIR mHealth uHealth. 2015;3(1):e27 25760773 10.2196/mhealth.3422 PMC4376132

[44] Antezana G, Bidargaddi N, Blake V, Schrader G, Kaambwa B, Quinn S, et al Development of an online well-being intervention for young people: an evaluation protocol. JMIR research protocols. 2015;4(2):e48 10.2196/resprot.4098 25929201 PMC4432225

[45] Kenny R, Dooley B, Fitzgerald A. Feasibility of "CopeSmart": a telemental health app for adolescents. JMIR mHealth uHealth. 2015;2(3):e22 10.2196/mental.4370. PMC4705022 26552425

[46] Chiang LC, Huang JL, Yeh KW, Lu CM. Effects of a self-management asthma educational program in Taiwan based on PRECEDE-PROCEED model for parents with asthmatic children. J Asthma. 2004;41(2):205–15. . 15115173 10.1081/jas-120026078

[47] Green L. The PRECEDE-PROCEED model of health program planning & evaluation [Internet]. c2014. Available: http://lgreen.net/precede.htm. Accessed 11 December 2014.

[48] Velsor-Friedrich B, Pigott T, Srof B. A practitioner-based asthma intervention program with African American inner-city school children. J Pediatr Health Care. 2005;19(3):163–71. 15867832 10.1016/j.pedhc.2004.12.002

[49] Hesse-Biber SN, Leavy P. The Practice of Qualitative Research: SAGE Publications; 2010.

[50] Holden RJ, Karsh B-T. The technology acceptance model: its past and its future in health care. J Biomed Inf. 2010;43(1):159–72. 10.1016/j.jbi.2009.07.002 PMC2814963 19615467

[51] Scheibe M, Reichelt J, Bellmann M, Kirch W. Acceptance factors of mobile apps for diabetes by patients aged 50 or older: a qualitative study. Med 20. 2015;4(1):e1 Epub 2015/03/04. . 25733033 10.2196/med20.3912 PMC4376102

[52] Doherty G, Coyle D, Matthews M. Design and evaluation guidelines for mental health technologies. Interact Comput. 2010;22(4):243–52.

[53] Jin BS, Ji YG. Usability risk level evaluation for physical user interface of mobile phone. HCC Sys Ind. 2010;61(4):350–63.

[54] Charmaz K. Constructing grounded theory: a practical guide through qualitative analysis / Kathy Charmaz. London: London: SAGE Publications; 2006.

[55] Radcliffe C, Lester H. Perceived stress during undergraduate medical training: a qualitative study. Med Educ. 2003;37(1):32–8. 12535113 10.1046/j.1365-2923.2003.01405.x

[56] Green J, Thorogood N. Qualitative methods for health research. 2nd ed Los Angeles: SAGE; 2009.

[57] Braun V, Clarke V. Using thematic analysis in psychology. Qual Res Psychol. 2006;3(2):77–101.

[58] Fereday J, Muir-Cochrane E. Demonstrating rigor using thematic analysis: A hybrid approach of inductive and deductive coding and theme development. Int J Qual Methods. 2008;5(1):80–92.

[59] Underwood B, Birdsall J, Kay E. The use of a mobile app to motivate evidence-based oral hygiene behaviour. Br Dent J. 2015;219(4):7 10.1038/sj.bdj.2015.660. 1707792818. 26315196

[60] Elias P, Rajan NO, McArthur K, Dacso CC. InSpire to promote lung assessment in youth: evolving the self-management paradigms of young people with asthma. Med 20. 2013;2(1):e1 Epub 2013/01/01. ; PubMed Central PMCID: PMCPmc4084766. 25075232 10.2196/med20.2014 PMC4084766

[61] Hebly P. Willingness to pay for mobile apps [Dissertation]. Rotterdam (Holland): Erasmus University Rotterdam; 2012.

[62] Lister C, West JH, Cannon B, Sax T, Brodegard D. Just a fad? Gamification in health and fitness apps. JMIR Serious Games. 2014;2(2):e9 10.2196/games.3413 . 25654660 PMC4307823

[63] Dolan B. The rise of the seemingly serious but "just for entertainment purposes" medical app [Internet]. 2014. Available: http://mobihealthnews.com/35444/the-rise-of-the-seemingly-serious-but-just-for-entertainment-purposes-medical-app. Accessed 20 January 2016.

[64] Phillips DC, Burbules NC. Postpositivism and educational research. Lanham: Rowman & Littlefield Publishers; 2000.

[65] Apple. App Store review guidelines [Internet]. 2015. Available: https://developer.apple.com/app-store/review/guidelines/. Accessed 12 April 2016.

[66] Android. Launch checklist [Internet]. 2016. Available: http://developer.android.com/distribute/tools/launch-checklist.html. Accessed 12 April 2016.

[67] OptumHealth. OptumHealth debuts OptumizeMe fitness app to help Microsoft(R) Windows Phone 7 users connect and compete for better health [Internet]. c2010. Available: http://www.businesswire.com/news/home/20101115005281/en/OptumHealth-Debuts-OptumizeMe-Fitness-App-Microsoft%C2%AE-Windows. Accessed 17 January 2015.

[68] Anderson K, Emmerton LM. The contribution of mobile health applications to self-management by consumers: review of published evidence. Aust Health Rev. 2015;In Press. 10.1071/AH15162 . 26681206

[69] Korhonen I, Parkka J, Van Gils M. Health monitoring in the home of the future. IEEE Eng Med Biol Mag. 2003;22(3):66–73. 12845821 10.1109/memb.2003.1213628

